# 

**Published:** 1905-04-01

**Authors:** 


					? tee hospital. " Cadbury's?the stanu I of highest purity.  LMTK.ee.  ^ AritlL 1ST,
Cadbury's rf* H fe* Cadbury's
KO DRUGS OR ALKALIES A /aML\ JM^s " For Purity and Nourishment there AAPAi
USED. wUvUA m nothing superior."?Uedical Mag. WwW^M
OS PIT A Lf
? - UJ -3=?   jJTHQMAS S HoSPTfArmmSS? I ?! ?I?U MM
LASCotr Western Infirmary ? ~ HOSTIJAL
No. 966. Vol. XXXVIII. [?Sfpfp?] Saturday,
April 1st, 1905.
^SubscriptionJerm.j pR,CE THREEPENCE.

				

